# Childhood asthma and mould in homes—A meta-analysis

**DOI:** 10.1007/s00508-024-02396-4

**Published:** 2024-07-11

**Authors:** Marton Kristof Varga, Hanns Moshammer, Oral Atanyazova

**Affiliations:** 1 ZPH, Department of Environmental Health, Medical University of Vienna, ZPH, Kinderspitalgasse 15, 1090 Vienna, Austria; 2 Karakalpakstan Medical Institute, 230100 Nukus, Uzbekistan

**Keywords:** Dampness, Conditions, Systematic review, Case-control studies, Cohort studies

## Abstract

Asthma is a multifaceted and multicausal disease. Childhood asthma is strongly influenced by genetic traits and is characterized by hyperreactivity of the airways so that also unspecific triggers including moulds can trigger an asthma attack. Therefore, it is undisputed that moulds in the home can cause asthma attacks in asthmatic children. It is, however, unclear if mould in homes also induce the development of asthma. Because more and more severe attacks in asthmatic children living in mouldy homes might speed up the diagnosis of asthma, cross-sectional studies are not well-suited to differentiate between mould as a causative or only as a precipitating factor. Cross-sectional studies show an increased asthma risk and poorer lung function in children living in mouldy homes. To better understand the causal role of mould in homes, a systematic review was performed with random effects meta-analysis focusing on cohort and case-control studies only.

We found 21 case-control and 11 cohort studies examining the association between mould at home and later advent of childhood asthma. According to the case-control studies, mouldy homes increase the risk of asthma by 53% (95 confidence interval [CI]: 42–65%) with no evidence of heterogeneity or publication bias. Risk estimates based on cohort studies were smaller with 15% (1–31%). The cohort studies also showed no publication bias but substantial heterogeneity (I^2^ = 60.5, *p* = 0.005). Heterogeneity could be partly explained by percentage of male children, age of participants, and publication year, but was not affected by study quality.

In conclusion, living in mouldy homes during childhood seems to increase the risk of later developing bronchial asthma.

**Supplementary Information:**

The online version of this article (10.1007/s00508-024-02396-4) contains supplementary material, which is available to authorized users.

## Introduction

Bronchial asthma is a heterogeneous disease characterized by paroxysmal episodes of respiratory symptoms (including wheezing, coughing, dyspnea, thoracic tightness) and is associated with bronchoconstriction, bronchial hyperreactivity and inflammation of the airways.

Bronchial asthma can occur for the first time at any age. The prevalence of bronchial asthma in childhood is approximately 10%, twice as high as in adulthood, although there are significant ethnic differences. According to the ISAAC studies [1–3], the highest disease rates are in Scotland, New Zealand, and Australia and the lowest in Asia and Eastern Europe. In terms of gender distribution, twice as many men as women suffer from asthma. According to a health survey from 2019 by Statistics Austria [4], 4.3% of Austrians aged 15 years and over suffered from bronchial asthma in the past year. The prevalence of asthma is higher in industrialized countries than in developing countries, with the prevalence converging in recent years. The rate is declining slightly in industrialized countries and increasing in developing countries [5].

In children, bronchial asthma is usually an allergic disease with asthma attacks triggered by various aeroallergens. Predisposition to type I allergic disease also dubbed “atopy”, has a strong genetic component, but as the prevalence of atopic disease has strongly increased in many parts of the world in the last decades, environmental causes are also deemed relevant in the development of atopic diseases including (allergic) childhood asthma [5, 6].

Among possible environmental causes, reduced training of the immune system in early life (“hygiene hypothesis”) is the key suspect [7–11]; however, also inhaled toxic and irritative pollutants are assumed to contribute to the increase in childhood asthma prevalence. Inhalative exposure can trigger asthma attacks in those already suffering from asthma or can worsen existing asthma symptoms. Asthmatic subjects, especially under insufficient treatment, are more vulnerable towards inhaled irritants. Therefore, in cross-sectional studies, higher symptom rates and poorer lung function are usually associated with a range of inhaled exposures, not only but also including living in damp and mouldy homes [5]. Nevertheless, cross-sectional studies are not well-suited for causal inference. Especially in the case of asthma, differentiating between an enhancement of symptoms in asthmatic people and an initiation of asthmatic disease is a challenge when the temporality of exposure and outcome cannot be assessed correctly. Therefore, in examining the causal relationship between irritative respiratory exposures and the onset of asthma, longitudinal studies, namely case-control and cohort studies, should be preferred.

Damp and mouldy homes constitute a frequent and serious setting exposing inhabitants to a range of irritative, toxic, and eventually also allergic substances. In Austria for example, repeated representative surveys [12] have reported that about 10% of all homes are affected by dampness and mould. In our own cross-sectional studies in schoolchildren, the reported prevalence of mould and moisture was even higher [13, 14]. Therefore, mould in homes lends itself as a model respiratory exposure to study long-term effects on the development of childhood asthma. According to the WHO review [15] the rate of dampness in Europe varies by country and study between 12.1% and 31.6%. The prevalence numbers in the USA are similar or even higher with percentages reported up to 50%. There are less data available from low-income countries but these indicate that dampness is a global problem although with a wide variation in prevalence.

The Institute of Medicine (IOM) committee [16], in a comprehensive literature review, found “sufficient evidence of an association between damp indoor environments and some upper respiratory tract symptoms, coughing, wheezing, and asthma symptoms in sensitized persons.” In the same review the evidence that dampness causes the (new) onset of asthma was deemed insufficient. Instead, the then existing evidence in that respect was rated as “limited or suggestive evidence of an association”.

Although the WHO review [15] concludes that “there is sufficient epidemiological evidence of associations between dampness or mould and (various health conditions)”, this association is not deemed sufficient proof of causality: “The epidemiological evidence is not sufficient to conclude causal relationships between indoor dampness or mould and any specific human health effect, although the findings of one strong epidemiological intervention study, in conjunction with the other available studies, suggest that dampness or mould exacerbates asthma in children.” This is mostly due to the fact that “the mechanisms by which non-infectious microbial exposures contribute to adverse health effects associated with indoor air dampness and mould are largely unknown.”

Two more reviews and meta-analyses on the associations between dampness and mould with asthma, allergies and respiratory symptoms have been published in 2011. Tischer et al. [17] concluded: “These findings suggest that home environments with visible mould and mould spore exposure increase the risk of allergic respiratory health outcomes in children. However, further investigations are needed to examine the effects of exposure to mould-derived components as the current literature is inconclusive.” Also, the review by Mendell et al. [18] comes to a similar conclusion: “Evident dampness or mould had consistent positive associations with multiple allergic and respiratory effects. Measured microbiologic agents in dust had limited suggestive associations, including both positive and negative associations for some agents. Thus, prevention and remediation of indoor dampness and mould are likely to reduce health risks, but current evidence does not support measuring specific indoor microbiologic factors to guide health-protective actions.”

Contrary to these conclusions, in our opinion it is not of primary interest if one single physicochemical or biological component causes respiratory symptoms and diseases in mouldy homes and can serve as a quantitative measure of exposure. As long as monitoring of bio-aerosols is still poorly standardized (bio-aerosols in mouldy homes consist of a very complex mixture of multiple irritating and toxic components), the presence of mould as reported by inhabitants or as assessed by expert judgment after visual inspection serves as good an exposure metric as any measurement [19–21]. Furthermore, several irritating inhaled exposures could cause the development of asthma in genetically susceptible children [22]. “Mouldy home” only serves as one example to underpin that assumption. To that end, reported mould is equivalent to any quantitative measurement. Hence, we set out to perform a systematic review and meta-analysis on the association between mould and asthma in children restricted to longitudinal studies, i.e. cohort-control and case-control studies.

## Methods

As the IOM [16] had already performed a thorough literature review, we restricted our search to papers published thereafter. A systematic search in PubMed, Scopus and Web of Science was performed using the search string “(home OR hous* OR dwelling OR residence OR residential OR residentially OR indoor* OR domicile* OR living OR unit* OR propert* OR build OR domestic OR environment* OR bedroom* OR living OR room) AND (fungi OR fungus OR mold* OR mould* OR fungal) AND (asthma* OR cough* OR wheez* OR dyspn*)”. We included papers published between the year 2000 and the end of the search which was 26 November 2022. In PubMed we set the filter “no case reports/reviews”. In Scopus, only title, abstract and keywords were searched. After removal of duplicates all titles and abstracts were screened for relevant papers. Relevant papers were read in full and data of interest were excerpted.

Included were studies based on original data and depicting the exposure-outcome relationship of mould in the home environment and the development of asthma in children and adolescents. The study population should include both genders in the age range between 1 and 19 years. Longitudinal observational studies, namely case-control and cohort studies with a risk estimate (odds ratio [OR]) and a narrowly defined confidence interval (90–95%) were included in the analysis, cross-sectional studies were excluded [23]. Furthermore, the studies should have a quantitative or qualitative measure of mould exposure and the outcome. The outcome asthma or asthma symptoms should be defined as follows: medically diagnosed asthma and/or persistent wheezing or repeated episodes of wheezing without a cold and/or asthma medication (bronchodilators/inhaled glucocorticoids) and/or evidence of reversible bronchial obstruction or hyperreactive bronchial system. When both outcomes were reported, prevalent asthma was chosen over asthma ever. Regarding exposure, binary metrics (mould yes/no) were preferred over ordinal (e.g.: no/minor/major) or continuous measures (e.g. concentration of some fungal indicator). This enabled a better comparison between the different exposure metrics. When exposure rating both by parents and by experts were reported, a conservative approach was chosen and the lower effect estimate was used. When multiple signs of mould were reported, visible mould was chosen over mouldy odour and over dampness or water damage and over composite indicators like “any of the above”. When the assessment was reported for different rooms in the household separately, the assessment for the child’s main living room was given precedence.

Studies that only equated exposure and sensitization to mould were excluded. Studies from specific populations where the focus was excessive indoor heating with fuels and studies in specific conditions/circumstances, such as after hurricanes, were also excluded. A Preferred Reporting Items for Systematic reviews and Meta-Analyses (PRISMA) flowchart was created to clearly illustrate the search process [24].

The following data were collected from the studies that met the inclusion criteria: name of the first author, year of publication, location of the study, type of study, age of participants at end point, duration of follow-up time for cohort studies, study size including number of cases and controls, factors according to which the ORs were adjusted if necessary, measures of exposure, definition of the outcome, risk estimates including confidence intervals.

When a study reported estimates for several outcomes or for several exposure metrics, usually a conservative approach was chosen and the lowest OR was used. When a cohort study examined the same population several times, the report using the most complete sample was selected.

The Newcastle-Ottawa scale (NOS) was used to assess the quality of the included non-randomized studies [25]. There were three aspects of the studies; selection, comparability and outcome/exposure were evaluated and up to nine stars were awarded depending on the risk of bias. Studies with seven or more stars have low bias, studies with more than four but fewer than seven stars have high bias. Studies with fewer than four stars have a very high bias and are not included in the meta-analysis. The most important factors used for comparability were gender and maternal allergy or asthma. Studies that controlled for gender received one star and studies that controlled for both factors received two stars.

Statistical analyses were performed using STATA 17 (StataCorp, 4905 Lakeway Drive, College Station, Texas 77845 USA). Meta-analyses were run separately for cohort and for case-control studies. The “meta” command in STATA expects a symmetric effect estimate. Therefore, log(OR) was calculated. For ease of graphical interpretation, the binary logarithm was used. Besides forest plots also funnel plots were produced to visually check for signs of publication bias. In the case of significant heterogeneity meta-regressions were also performed to look for possible sources of heterogeneity. In these regressions the logarithms of the odds ratios were regressed against possibly influential factors weighted by study size. That part of the work was performed within the framework of a diploma thesis and the results have been published in German [26]. For the current paper, the original collection of studies was re-evaluated and updated:

In addition to the papers published since the year 2000, also the case-control and cohort studies on children reported by IOM were included in the meta-analyses. As these older studies were not excerpted in detail, but only the effect estimates were obtained from the IOM review, these studies could not be included in the meta-regression.

## Results

**Fig. 1:**
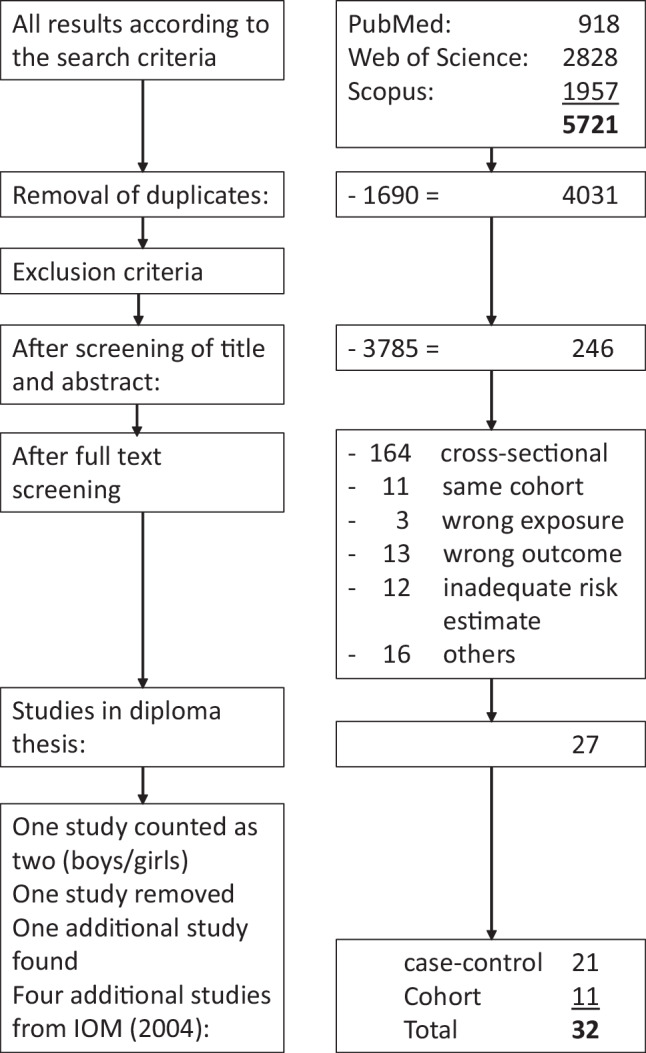
Prisma flowchart

The PRISMA flowchart (Fig. 1) shows that a total of 5721 studies were found in the search. After removing duplicates (1690), 4031 studies were screened by title and abstract. Finally, 27 of the 246 studies examined in full text were included in this meta-analysis. Over 60% of the studies examined in full text were excluded due to the study design (cross-sectional studies). To these 27 studies, 4 additional studies were added from the IOM [16] review: 2 cohort studies [27, 28] and 2 case-control studies [29, 30]. Oie et al. [31], also from the IOM [16] review, was not included, because this nested case-control study was based on the same cohort as the one from Nafstad et al. [28], which had the larger data base. The study by Infante-Rivard et al. [32], although reported by IOM [16] did not use mould but humidifier use as an exposure metric.

In addition, we excluded the cohort study by Cox et al. [33]. That study had first constructed a “mould index” rating different bacteria and fungi by their association with symptoms of wheeze and had then calculated the OR of that index for asthma. This resembled circular reasoning. Still, that cohort study was interesting as it observed children of this birth cohort at the age of 7 years and 12 years. It demonstrated that the OR was higher at 12 years old (OR 1.33; 95% CI 1.12, 1.53) than at 7 years old (OR 1.22; 95% CI 1.06, 1.40). Another cohort study [34] was later detected and included.

Table 1 (cohort studies) and Table 2 (case-control studies) show an overview of the included studies. A total of 11 (birth) cohort studies and 21 case-control studies, 15 of which were nested, were analyzed, 10 studies were from Europe, 8 from North America, 9 from Asia, and 1 from New Zealand. The country of origin was not reported for the 4 studies excerpted from the IOM [16] review. In total, there were 2 very large (*n* ≥ 10,000), 9 large (*n* ≥ 1000), 19 medium-sized (*n* ≥ 100) and 3 small studies (*n* < 100). In the last list, the study by Wen et al. [35] counted as “very large” for boys and “large” for girls.

**Table 1:** Summary of cohort studies

Study	Year	Country	Age at follow-up (years)	Sample size	Exposure	Asthma definition	NOS
[38]	2022	Japan	3	60,529	Questionnaire—visible mould	Questionnaire—doctor diagnosed asthma	6/9
[33]	2022	USA	12	112	Dust sample—qPCR (bacteria and fungi)	Questionnaire—various endpoints. Excluded because of circular exposure definition	7/9
[37]	2017	Sweden	16	3798	Questionnaire—visible mould, odour, dampness (number of indicators)	Questionnaire—various endpoints, symptoms and therapy	6/9
[39]	2016	Finland	6	93	Inspection for visible mould	Questionnaire—doctor diagnosed asthma, symptoms, therapy	7/9
[35]	2015	Taiwan	5	19,192	Questionnaire—visible mould	Questionnaire—doctor diagnosed asthma	6/9
[40]	2015	Finland	6	398	Inspection for visible mould	Questionnaire—doctor diagnosed asthma, symptoms, therapy	7/9
[34]	2014	USA	13	408	Questionnaire—visible mould	Questionnaire—doctor diagnosed asthma, symptoms, therapy	–
[41]	2014	Canada	11–14	422	Dust sample—high beta-Glucan	Pediatric diagnosis based on Canadian Asthma Consensus guidelines	7/9
[42]	2005	Finland	7–13	1919	Questionnaire—visible mould	Questionnaire—doctor diagnosed asthma	6/9
[28]	1998	–	3–5	1085	Questionnaire—dampness or visible mould	–	–
[27]	1997	–	5–9	925	Questionnaire—visible mould	–	–

**Table 2:** Summary of case-control studies

Study	Year	Country	Type	Age (years)	*N* cases/controls	Exposure	Asthma definition	NOS
[43]	2022	China	–	3–14	160/247	Questionnaire—visible mould	Hospital diagnosed asthma	5/9
[44]	2021	China	–	3–12	608/839	Questionnaire—visible mould	Hospital diagnosed asthma	6/9
[45]	2018	New Zealand	–	1–7	150/300	Questionnaire—visible mould	Questionnaire—symptoms	8/9
[46]	2017	Canada	Nested	2–7	89/108	Dust sample—fungal spores	Questionnaire—doctor diagnosed asthma, symptoms	7/9
[47]	2014	USA	Nested	7	13/28	Questionnaire—visible mould	Questionnaire—doctor diagnosed asthma, symptoms, therapy	5/9
[36]	2013	France	Nested	9–15	44/51	Air sample for MVOCs	Questionnaire—doctor diagnosed asthma, symptoms, therapy	8/9
[48]	2012	Taiwan	Nested	1–7	188/376	Questionnaire—visible mould	Questionnaire—doctor diagnosed asthma	7/9
[49]	2011	USA	Nested	≤17	50/49	Air sample for fungal spores	Questionnaire, various criteria	8/9
[50]	2011	Taiwan	Nested	12–14	193/386	Questionnaire—visible mould or odour	Questionnaire—doctor diagnosed asthma, symptoms	7/9
[6]	2011	Chile	–	6–15	188/294	Questionnaire—visible mould/dampness	Hospital diagnosed asthma	6/9
[51]	2010	USA	Nested	≤17	530/882	Questionnaire—visible mould or odour	Questionnaire—doctor diagnosed asthma	5/9
[52]	2009	Sweden	Nested	1–6	121/202	Questionnaire—visible mould or odour	Questionnaire—symptoms	8/9
[53]	2007	Finland	–	1–7	121/241	Inspection on mould	Hospital diagnosed asthma	9/9
[54]	2005	NL and DE	Nested	7–8	600/601	Questionnaire—visible mould/dampness	Questionnaire—symptoms	5/9
[55]	2004	Sweden	Nested	10 LJ	144/144	Questionnaire—visible mould/dampness	Hospital diagnosed asthma	6/9
[56]	2003	Palestine	Nested	6–12	237/252	Questionnaire—visible mould	Questionnaire—symptoms	5/9
[57]	2003	UK	Nested	9–11	193/223	Inspection on mould	Questionnaire—symptoms	5/9
[58]	2002	China	Nested	6–10	403/806	Questionnaire—visible mould	Questionnaire—doctor diagnosed asthma	7/9
[59]	2001	Canada	Nested	5–19	592/443	Questionnaire—visible mould	Questionnaire—doctor diagnosed asthma, symptoms, therapy	4/9
[29]	1998	–	Nested	2	251/251	Questionnaire—dampness	Bronchial obstruction	–
[30]	1998	–	–	3–15	86/86	Questionnaire—dampness	–	–

*NOS* Newcastle-Ottawa Scale; *DE* Germany, *NL* Netherlands

The definition of exposure differed between studies, with some studies also using multiple methods. For example, in two studies [33, 36] two methods were combined to calculate the OR. In one study [35], the OR was stratified by gender and accordingly both ORs were used (counting as two studies). The study found no significant effect in boys (OR: 1.01) and therefore did not include “mould” in the final multivariate analysis. This was the only study where we had to use the unadjusted OR. Although from all other studies adjusted ORs were abstracted, when possible we did not use the results of these models that also controlled for sensitization towards fungal allergens, because this was not seen as a confounder but a mediator. Of the studies seven only asked about visible mould, five studies assessed the presence of mould spots and/or moisture and seven studies assessed the presence of mould spots and/or the smell of mould using parental questionnaires. A building inspection was performed in eight studies. Additionally, dust samples were taken in six studies and air samples were taken in two studies, but usually also binary exposure variables were constructed from these continuous data. Thacher et al. [37] asked about visible mould, mouldy odour, and signs of dampness and reported effect estimates for one, two, or three criteria met (compared to none) to demonstrate a significant dose-response-association (ORs: 1.16, 1.37, 1.73). In that instance, we included the “middle” estimate of 1.37 (95% CI 1.01, 1.86).

The definition of asthma was also very variable in some cases; in two studies the diagnosis was validated using a methacholine test and in one using an inhalation provocation test. In four case-control studies, asthmatics were recruited directly from the hospital where they were treated or received their diagnosis, 11 studies asked about doctor-diagnosed asthma, and 7 of them also asked about an additional factor. In 1 study, asthma was defined as doctor-diagnosed asthma or wheezing without a cold in the past 12 months, 3 studies asked about multiple episodes of wheezing, 1 study asked about wheezing in the last 12 months and 1 asked about wheezing in the last 12 months or persistent cough for approximately 3 months and 1 asked about multiple episodes of wheezing or at least one episode of wheezing in in the last 12 months associated with the use of inhaled glucocorticoids. In one study, asthma was defined as wheezing with inhaled bronchodilator use. In two studies, one of three or two of seven of the factors had to be met for signifying a child with asthma. These differences could have introduced some variation between the effect estimates, but generally we assume that the relative risks for outcomes of the same class will not differ substantially.

**Fig. 2:**
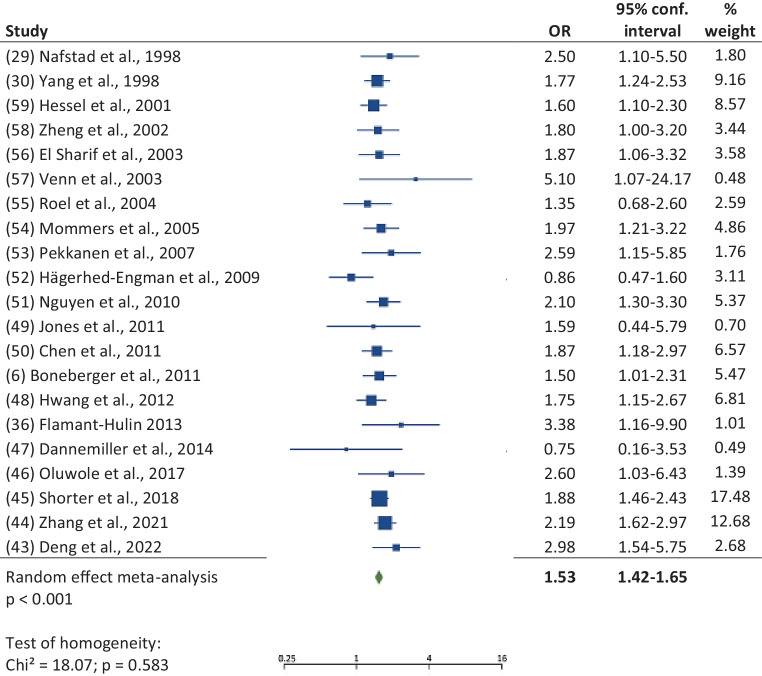
Forest plot of case-control studies

In a meta-analysis 21 case-control studies revealed a significant (*p* < 0.001) effect of early mould exposure on asthma development (Fig. 2): OR 1.53; 95% CI 1.42, 1.65 without any sign of heterogeneity (*p* = 0.583). The funnel plot (supplementary figure S1a) gave no indication of publication bias.

**Fig. 3:**
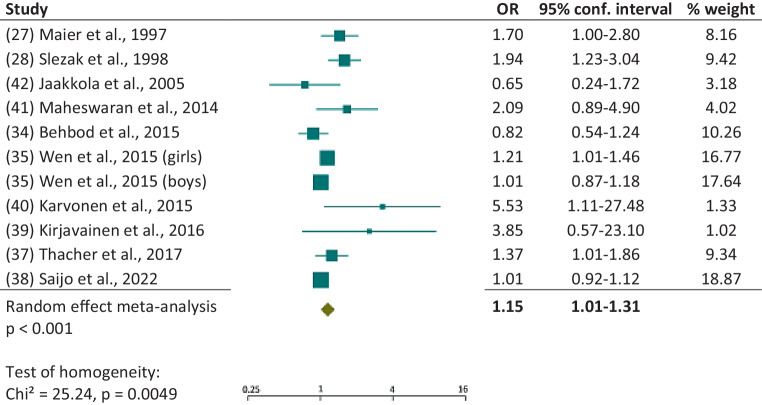
Forest plot of cohort studies

The 11 cohort studies also gave a significant effect (*p* = 0.038), albeit a weaker one (Fig. 3): OR 1.15; 95% CI 1.01, 1.31, but with clear evidence of heterogeneity (*p* = 0.005). The funnel plot (figure S1b) gave no hint of publication bias either.

Because of the clear signs of heterogeneity in the cohort studies, factors accounting for it were sought in meta-regression. At first, cohort studies were analyzed separately again. As expected, none of the tested factors approached any significance in case-control studies (most *p* > 0.5) but in cohort studies, the following factors demonstrated at least a tendency to increase the OR when examined together: percentage of boys (*p* = 0.088), age at diagnosis (*p* = 0.07), and publication year (*p* = 0.081). As these factors also displayed the same positive sign in case control studies, although not significant, case-control and cohort studies were pooled in the next step and the binary indicator of “cohort study” was added to this regression model. In this model, all factors reached significance (Table 3). The NOS (or a binary bias indicator derived thereof) never approached significance. For cohort studies, a larger NOS (indicating better study quality or less bias), led to a somewhat larger OR, although this effect did not reach significance.

**Table 3:** Results of linear meta-regression on the binary logarithm of the OR, weighted by study size

Factor	Coefficient	95% confidence interval
Cohort (versus case-control)	−0.8136	−1.1163, −0.5109
Percent male participants	0.0044	0.0003, 0.0086
Age at inclusion	0.0346	0.0066, 0.0626
Publication year	0.0280	0.0044 0.0517

Again, in the combined study set, controlling for “cohort” status, exposure assessed by visual observation displayed a lower OR, although this effect was not significant (*p* = 0.108). There were not enough studies included that based the exposure judgement on odour, as we had decided a priori to prioritize visual assessments. Exposure assessed by parents led to higher ORs than that assessed by experts (*p* = 0.001) but any type of quantitative measurement as compared to parents’ or experts’ judgement did not fare differently (*p* = 0.411).

## Discussion

Cross-sectional studies consistently indicate an increased development of bronchial asthma in children after early life exposure to a mouldy home. The relative risk was comparatively high (with about 1.5). Given the high frequency of mouldy homes (about 10%) and the high prevalence of asthma in children (also around 10%), this would mean that in 10% of homes another 5% of the children would develop asthma because of the exposure. In the population, this would translate to 0.5% of all children suffering from asthma because of this exposure. From a public health point of view, considering the individual and the general harm caused by bronchial asthma, that number is very high.

Usually, cohort studies are perceived as the gold standard in observational studies. Surprisingly, in the case of mould exposure and asthma development, cohort studies not only found a much weaker effect, but also were much less consistent overall with very strong evidence for heterogeneity. While case-control studies suffer from retrospective exposure assessment that might be affected by reporting bias, this is not the case in well-conducted cohort studies, where the exposure assessment is performed before the outcome and therefore cannot be affected by it. Nevertheless, cohort studies can suffer from loss to follow-up, which could also be non-differential. To ensure robust statistics, cohort studies either depend on a large number of participants or must be restricted to relatively frequent outcomes. While bronchial asthma by itself is not a very rare disease, the combination between asthma and mould is still so rare that it might make cohort studies difficult. A viable and often used solution would be to focus on specific vulnerable subgroups, most often creating a (birth) cohort of children with a family history of asthma. This definition of vulnerability does likely vary between studies. Only one of the cohort studies [34] included explicitly targeted children with family history of allergic disease or asthma; however, also for other cohort studies it seems plausible that parents with some personal interest in the studied outcome would be more willing to participate leading to a bias towards higher genetic risk. It is not clear if the relative risk from mould is the same for vulnerable groups and for the general public. This leads to the question if the relative risks from genetics and from environment are additive or multiplicative in nature. A smaller relative risk in a vulnerable group could still translate into a higher absolute risk. If indeed participants in cohort studies tend to represent persons with a higher genetic risk more often than in the average population, this could lead to a reduced relative risk for specific environmental exposures.

The same mechanism might be at play with gender differences. As children, boys have an increased prevalence of asthma [60]. Yet, in the large study by Wen et al. [35], no relevant risk was found for boys (OR = 1.01), but a significant one for girls (OR = 1.21). Therefore, we assumed that studies with a higher percentage of boys would give a smaller OR. Because in cohort studies, asthma would be more often diagnosed in boys, this might explain the smaller OR found in cohort studies but in the meta-regression the reverse was true and a higher percentage of boys even led to a higher OR. For this finding, we have no clear answer. Also, the lower effect estimates reported in cohort studies calls for further elucidation.

Sometimes people report their own experiences of living in mouldy flats and confirm that living in a mouldy flat is clearly recognizable and will not be forgotten. Therefore, erroneous reporting and hence a relevant reporting bias seems highly unlikely and assessment of mould exposure by inspection and lay assessment does correlate well with measurement data [19]. Indeed, the exposure “mouldy home” was chosen as an example of early life exposure to a multitude of irritating substances exactly because it is so easily recognizable. This is not the question if a certain component (e.g., a certain microbial volatile compound, a spore of a certain fungal species, or some chemical part of the fungal structure) “causes” asthma. Many irritating substances, partly with allergenic and/or toxic properties, partly strong stimulants of the immune system, are increased in mouldy homes. A simple inspection should suffice to declare a situation to pose a risk to the inhabitants. Indeed, in the meta-regression, sophisticated methods of quantification and measurement did not fare better than simple inspection. Regarding inspection, parent-reported mould gave higher ORs than experts’ reports. Parents’ reports might be somewhat biased because a coughing and wheezing child might make them more aware of any housing problems; however, parents might also just know better about the real housing conditions while the impression of an external expert might be unduly influenced by chance findings that do not reflect the longer term situation.

While study quality or risk of bias as assessed by the Newcastle-Ottawa Scale did not affect the size of the effect, publication year did with later studies tending to find stronger effects. It can be hypothesized that more recent studies were less subject to non-differential errors regarding exposure and/or outcome. Higher age at study inclusion was associated with stronger effects. In this respect it might be interesting to note that average age was strongly correlated with follow-up duration in cohort studies (R = 0.73). The effect of average age might indicate a slow development of the disease that causes a considerable latency between exposure and final diagnosis. A shorter follow-up time might be affected by reverse causality: Even before children are diagnosed with asthma, their respiratory symptoms might lead the parents to seek a better home. Therefore, the observation of a stronger effect after a longer latency clearly supports the notion of causality.

## Conclusion

Mould and dampness in homes serve as a relevant example of an inhaled irritant exposure of children. Early childhood exposure increases the risk of bronchial asthma later. Cohort studies and even more so case-control studies demonstrated a significant association. Heterogeneity of the effects seen in cohort studies are best explained by publication year and age of the children. There was a tendency towards stronger effect estimates in studies with a lower risk of bias. Taken together, these findings support the notion that the association found is causal.

## Supplementary Information

Figure S1: Funnel plots: (A) case-control, (B) cohort studies
