# Supplementary figures and images for: Childhood asthma and mould in homes—A meta-analysis
Source: Wien Klin Wochenschr. 2024 Jul 11;137(3-4):79–88. doi: 10.1007/s00508-024-02396-4 (PMC11794369; doi:10.1007/s00508-024-02396-4)

Figure S1: Funnel plots: (A) case-control, (B) cohort studies


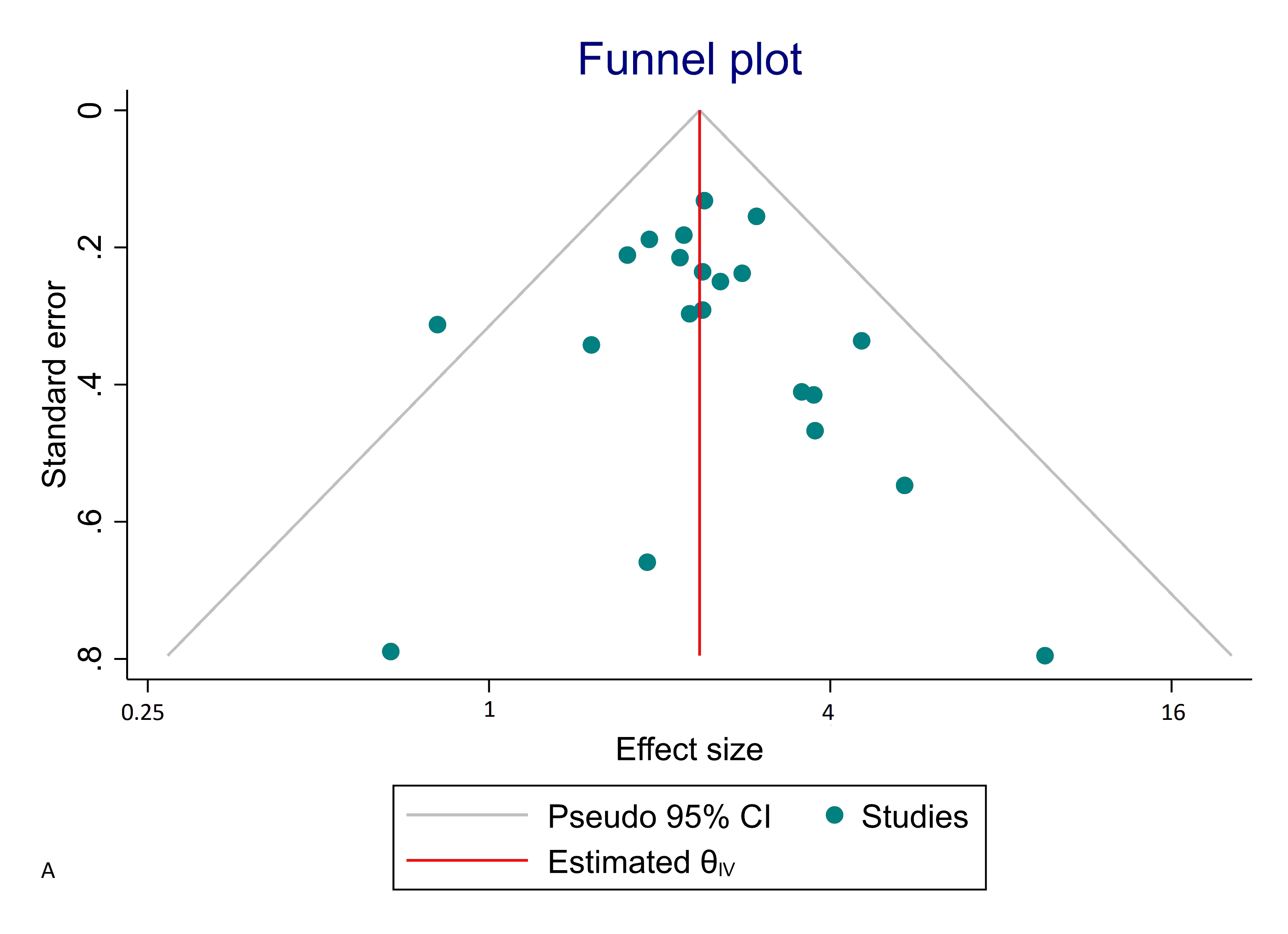


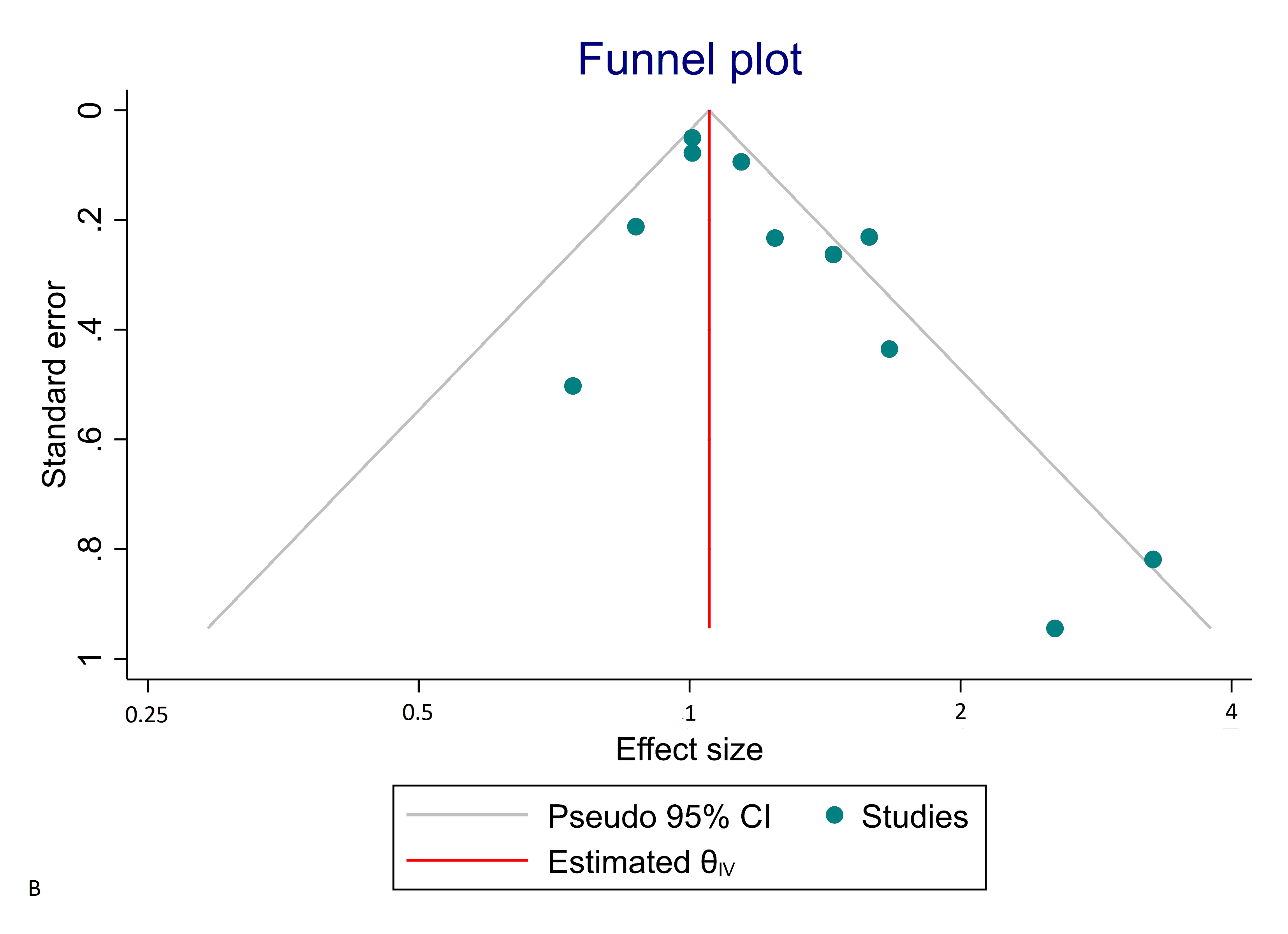

Supplement: Supplementary file 1 — Figure S1: Funnel plots: (A) case-control, (B) cohort studies [file 508_2024_2396_MOESM1_ESM.docx]
